# ‘My cousin said to me . . .’ Patients’ use of third-party references to facilitate shared decision-making during naturally occurring primary care consultations

**DOI:** 10.1177/13634593231188489

**Published:** 2023-07-30

**Authors:** Olaug S Lian, Sarah Nettleton, Huw Grange, Christopher Dowrick

**Affiliations:** UiT The Arctic University of Norway, Norway; UiT The Arctic University of Norway, Norway; University of York, UK; UiT The Arctic University of Norway, Norway; University of Liverpool, UK

**Keywords:** narrative analysis, patient-physician relationship, primary care

## Abstract

In this paper, we explore the ways in which patients invoke third parties to gain decision-making influence in clinical consultations. The patients’ role in decision-making processes is often overlooked, and this interactional practice has rarely been systematically studied. Through a contextual narrative exploration of 42 naturally occurring consultations between patients (aged 22–84) and general practitioners (GPs) in England, we seek to fill this gap. By exploring how and why patients invoke third parties during discussions about medical treatments, who they refer to, what kind of knowledge their referents possess, and how GPs respond, our main aim is to capture the functions and implications of this interactional practice in relation to decision-making processes. Patients refer to third parties during decision-making processes in most of the consultations, usually to argue for and against certain treatment options, and the GPs recognise these utterances as pro-and-contra arguments. This enables patients to counter the GPs’ professional knowledge through various knowledge-sources and encourage the GPs to target their specific concerns. By attributing arguments to third parties, patients claim decision-making influence without threatening the GPs’ authority and expertise, which their disadvantaged epistemic position demands. Thereby, patients become able to negotiate their role and their epistemic position, to influence the agenda-setting, and to take part in the decision-making process, without being directly confrontational. Invoking third parties is a non-confrontational way of proposing and opposing treatment options that might facilitate successful patient participation in decision-making processes, and so limit the risk of patients being wronged in their capacity as knowers.

## Introduction

Calls to respect patient autonomy and practise patient/person-centred care have been amplified in western healthcare systems, especially during the last decades (Dowrick, 2018; Lewis and Holm, 2022). A key component of this ideology is the ideal of shared decision-making (Barry and Edgman-Levitan, 2012). When decisions are shared, actions are undertaken in collaboration with patients, not just on their behalf. Acknowledging the autonomy of the help-seeking person has normative significance in the sense that it is a protection against illegitimate paternalistic clinical interventions (Dowrick, 2018; Lewis and Holm, 2022).

Although defined and argumentatively supported in different ways (Bomhof-Roordink et al., 2019), shared decision-making (SDM) means that patients and clinicians work together to reach joint decisions about further actions through a collaborative process where decision-making power is shared, and patients’ values respected (Elwyn, 2011). This requires actions from both parties: clinicians must be respectful of and responsive to patients’ experiences, needs and values (Epstein and Street, 2011), and patients must engage actively (Barry and Edgman-Levitan, 2012). However, because the onus of achieving SDM is usually placed on healthcare professionals (Bomhof-Roordink et al., 2019), the patients’ role is easily overlooked, and their engagement remains under-investigated (Street, 2021).

In this paper, we explore the ways in which patients contribute to SDM during naturally occurring consultations with general practitioners (GPs) through a narrative analysis of decision-making processes. Our exploration is limited to the ways in which patients make use of third-party references while discussing medical treatment options with their GPs. By a third party we mean a source to whom information is attributed (Drew, 1991), a source that is ‘outside’ of the interaction. Patients and GPs are first and second parties; the third party is anyone else (a person, a group of persons, or people in general) not directly involved in the interaction, but indirectly included through a reference. Usually, third-party references are part of mitigation strategies; they make speech acts less outright and more indirect. By allowing a third party to indirectly enter the conversation and mediate arguments, patients justify performing these utterances by attributing them to someone else, or at least leaning on others to substantiate their views.

Empirically, our study is based on verbatim transcripts of 42 naturally occurring consultations with GPs in England, sourced from a corpus of 212 consultations from the *One in a Million* archive at the data repository of the University of Bristol, UK (Table 1). By narratively exploring the moment-to-moment unfolding of chains of speech acts in dialogues between patients and GPs, we seek to gain knowledge about *how* and *why* patients invoke third parties during decision-making processes, *who* they refer to, *what* kind of expertise they draw on, and *how* GPs respond. Our main aim is to capture the principal functions and implications of this interactional practice in relation to SDM.

**Table 1.** One in a million: Primary care consultations archive.^a^

Type of study	A prospective observational study containing an initial dataset, archived at the data repository of the University of Bristol, UK.
Data material	327 film- or audio-recorded and verbatim transcribed naturally occurring GP consultations collected between 2014 and 2015 in 12 National Health Service practices in and around the City of Bristol. Consultations took place between adult patients (aged 18–96) and 23 different GPs. A total of 300 patients gave informed written consent for their data to be accessed and reused by ‘other researchers, subject to specific ethical approval’. From this sample, we received 212 consultations (all consultations related to cardiovascular, musculoskeletal, psychological, digestive, endocrine/metabolic, neurological and general conditions). The dataset also includes patient records; longitudinal patient pre- and post-consultation survey data; sociodemographic data of patients and GPs and GP practice data.
Funding	The One in a Million study was funded by the National Institute for Health Research (NIHR) School for Primary Care Research (208) and the South West GP Trust, and received ethics approval from South West – Central Bristol Research Ethics Committee (ref.: 14/SW/0112).

a Barnes, 2017; Jepson et al. (2017).

Research about the role of patients’ third-party references in discussions about treatment options during clinical consultations is scarce. Patients’ use of third-party references has been studied in relation to doctor-patient talk more generally (Nguyen and Austin, 2018), and occasionally mentioned in studies related to diagnostic issues (Ainsworth-Vaughn, 1998; Robson and Lian, 2016) and causal explanations (Gill, 1998; Gill and Maynard, 2009). The role of information from the internet in patients’ interactions with doctors has also been studied (Stevenson et al., 2021). Occasional examples of third-party references from patients in relation to treatment options have been reported, for instance ‘my kids was wondering if you thought I should . . .’ (Ainsworth-Vaughn, 1998: 40), but, to the best of our knowledge, no one has systematically studied patients’ use of references to third party sources during decision-making processes in naturally occurring clinical consultations.

The most relevant previous study is from primary care in a Vietnamese setting. After studying patients’ and doctors’ third-party references in all consultation phases, Nguyen and Austin (2018) conclude that patients invoke third parties to circumvent troublesome administrative requirements, obtain a preferred treatment, receive a health-related service, give reasons for selecting healthcare providers, or challenge the doctor’s expertise. They interpret these findings as related to the Vietnamese culture:

We suggest that doctors and patients are particularly inclined to invoke relatives-plus-medical professionals as third parties because of two social forces within Vietnamese culture: collectivism and social status. [. . .] More broadly, our findings indicate that medical communication is not invariant across cultures, but can be shaped by culture-specific forces. (Nguyen and Austin, 2018: 713)

With empirical data from a European setting, we reflect on issues raised here regarding the interaction between national, culture-specific forces and institutional forces created by modern biomedicine and contemporary health systems.

## Theoretical perspective

We interpret the interaction between patients and doctors as embedded in a social field constituted by a set of interconnected, complementary and asymmetrical social positions (Bourdieu, 1989). Actors who hold these positions face institutionalised normative structures that promote and counteract certain practices. When entering their positions in this field, actors become responsive to the pre-set repertoire of culturally shared norms and values they are expected to act upon. These informal and taken-for-granted rules of conduct are tacitly claimed by each party, and they create a kind of ceremonial order (Strong, 1979). Actors can choose to honour, invert or disregard them, but the interaction is, nonetheless, played out between participants who know – in the words of Bourdieu – ‘the immanent rules of the game’ (Bourdieu and Wacquant, 1992: 99). These rules include discursive frames that define ‘institutionally specific discursive opportunities’ (Snow, 2008: 6), and mark the limits of what Butler (1997) refers to as acceptable speech. Norms of acceptable speech demarcate ‘the line between the domains of the speakable and the unspeakable’ (Butler, 1997: 356). It is against this background that chains of speech-acts in a dialogue must be understood.

Within a social field, which in our case is the clinical consultation, negotiations occur on both a positional and an individual level. On a positional level, it is the two *positions* of patient and doctor that are negotiated (Freidson, 1970). This does not imply that individuals lack agency, nor that they invariably have divergent goals, but that doctors and patients negotiate from specific positions that they represent in the here and now of the consultation. On an *individual* level, negotiations are performed through verbal exchanges of speech-acts between individuals who may or may not comply to ‘the immanent rules of the game’. While doing so, they need to ‘navigate the structural constraints and imperatives that their contradictory locations give rise to’ (Wainwright et al., 2015: 19). Initiatives to replace paternalistic communication patterns with patient-cantered care support ‘the engaged patient’ (Timmermans, 2020). Nevertheless, their engagement needs to be in line with the institutionalised normative structures of the clinical consultation.

In a clinical consultation, the deployment of and reliance on epistemic resources are normatively organised in a hierarchy (Stivers et al., 2011). Patients are entitled to knowledge about their problems by virtue of experiencing them (Heritage, 2011), but in the field of clinical interaction the experiential position of patients is subordinate to the authoritative epistemic position of GPs. Doctors have an institutionally based authority-position which means that, in the end, they are the ones who largely determine the rules of the game (Stokes et al., 2006). In England, GPs have the right to remove patients from their lists when there is ‘disagreement between the practice and patient’ (British Medical Association, 2020), which means that, in our case, the list of possible sanctions includes total exclusion.

Our theoretical orientation ensures that we look at the data through a contextual lens appreciating the social field, the social positions, and the power dynamics of the interactions.

## Data and method

Our study is based on a contextual narrative analysis of verbatim transcripts of 42 naturally occurring GP consultations, sourced from a corpus of 212 consultations from the *One in a Million* archive (Table 1).

### Data material

**Figure 1.**
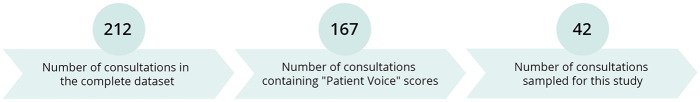
Sample selection.

Based on a data-grounded thematic coding of all 212 cases in NVivo version 12.4 (Lian et al., 2021), we found particularly active participation by patients in 167 consultations. Of these 167 consultations, we selected a sample of 42 (25%) for further in-depth analysis (Figure 1, Table 2). To ensure a sample with actively engaged patients, we first sampled all consultations with more than nine ‘patient voice’ utterances, that is, questions, suggestions, opposition and opinions (13 consultations). We then added 29 consultations with 1–9 ‘patient voice’ scores based on patient and GP characteristics. As a result, our sample (Table 2) contains patients who are more actively engaged than patients in the remaining consultations, but it mirrors the original dataset of 212 consultations in relation to patient age range (18–92 years); patient gender distribution (64% women and 36% men); amount of patients who met what patients defined as their ‘usual’ GP (about two thirds), and amount of consultations performed by women GPs (about two thirds). We used information from patient records to support our interpretations of the consultation transcripts.

**Table 2.** Consultations (*n* = 42).

Patient gender	Range	Average
Patient age		
Women (*n* = 26)	23–84	57.9
Men (*n* = 16)	22–78	56.7
GP age		
Women (*n* = 26)	34–62	45.8
Men (*n* = 16)	32–58	44.9
Consultation length (minutes)		
Women (*n* = 26)	5–26	15
Men (*n* = 16)	7–20	15
‘Patient Voice’ scores		
Women (*n* = 26)	1–19	6.9
Men (*n* = 16)	1–16	7.4

### Data analysis

During the first stage of our analysis, we identified all dialogue sequences containing third-party references from patients and searched for patterns across all 42 consultations, before developing a classification based on two main criteria: (1) rhetorical function (pro-et-contra arguments), and (2) type of referent (family members, friends, other health professionals and public sources). After classifying dialogue extracts based on this scheme, we explored the dominant patterns. To make sense of dialogue sequences, we related them to all remaining parts while treating each consultation as a narrative. During our main analysis, we narratively explored the unfolding of each consultation individually, emphasising *what* was uttered (content), *how* it was uttered (form) and *by whom* (speaker). We interpret our findings in relation to the discursive frame of the social field; the clinical consultation.

Our empirical data consist of dialogical data, where meanings emerge through reciprocal exchange. Every utterance is ‘either a statement establishing the next speaker’s words as a reply, or a reply to what the prior speaker has just established’ (Goffman, 1992: 78). To preserve context and meaning, while also capturing the ongoing dynamics of the interactional flow, we mainly worked with dialogue sections. Our focus on the interactional dynamics is in keeping with Riessman’s (2008) performative narrative analysis. By quoting long extracts and analysing components in light of the whole, we respect the integrity of the narrative.

### Ethics

The One in a million study received ethics approval from South West – Central Bristol Research Ethics Committee (ref.: 14/SW/0112). Our study received ethics approvals from the National Health Service (Research Ethics Committee reference 18/WM/0008; Integrated Research Application System project ID 232578), and Bristol Data Repository clearance from the Data Access Committee. All data were anonymised upon receipt, and there was no contact with study participants. The dataset was stored on a password-protected site at the University of York, UK, accessible to first and second author only.

## Results

Patients of all ages (from 22 to 84) referred to third parties, once or several times, in 24 of the 42 consultations (13 of 26 women and 11 of 16 men). A total of 26 different utterances are observed, divided according to three main types of referents: (1) a ‘displaced author’ (someone to whom the patient attributes a suggestion, request or challenge, displacing responsibility for an utterance away from themselves) (Ainsworth-Vaughn, 1998: 181), (2) a ‘proxy patient’ (someone in a similar situation to themselves), and (3) a public source (various mass media sources). Based on their dialogic contexts, we interpret these utterances as pro-and-contra arguments made during decision-making processes (with contra arguments the more prevalent). Both types of arguments are ambiguous in the sense that they are not necessarily proposals and rejections; they could also be a kind of conversation starter (and, thereby, serve to set the agenda for what to discuss). Nevertheless, they serve as arguments either for or against a treatment option. We structure our data presentation in relation to two main dimensions: type of argument (pro-et-contra) and type of referent (‘displaced author’/‘proxy patient’ and public sources).

### Patients use third-party references as pro-arguments

#### References to ‘displaced author’/‘proxy patient’

While using a third-party source to *indirectly* suggest a medical treatment, a man in his early 40s refers to a school friend who used Prozac in a consultation with his ‘not usual’ GP. Because of the side-effects he is experiencing, he wants to explore alternatives to his current antidepressant medication (fluvoxamine):

Case 1:

P: I haven’t tried Prozac yet.

GP: There are a bunch we can try. The downside to them is that you have to come off one, start the next one and it takes a few weeks to feel better on the next one. It is not worth doing three weeks on one you really want to do it for a couple of months to properly see if it is going to make a difference. Like I said there just isn’t a quick fix answer.

P: A school friend of mine has, and it has just sorted her out after years.

GP: Sertraline does seem to be really good for many people.

P: It did work at first and then I felt a little bit shouty, but maybe ___something else.

GP: If we are struggling, it is definitely one we could try again in the future, but there are a few others we could try as well.

Citing the case of a school friend (a ‘proxy patient’) appears to imply a proposal to move to an alternative antidepressant (Prozac). It serves as a non-constraining indirect request for the GP to consider whether Prozac would also be appropriate for him. The patient does not put the GP in a position where he is obliged to answer because, strictly speaking, it is not posed as a proposition, but the GP interprets it as such and responds accordingly by suggesting an alternative: sertraline. When the patient expresses reservations about sertraline – medication he has tried in the past – the GP postpones the debate to sometime in the future, and explains why. The patient record reads: ‘stay on reduced dose (50 mg) of fluvoxamine for a couple of weeks and then review, if still bad side-effects, then to discuss coming off with a view to trying an alternative’. In the record, she also notes that she is not his ‘usual’ GP, which might partially explain her wait-and-see strategy.

Patients rarely express the purpose of their third-party references explicitly, especially not in relation to pro-arguments. A woman in her early 50s who meets her ‘usual’ GP is a rare exception:

Case 2:

P: The back thing is just getting worse and worse and worse. A friend of mine did say, ‘Ask if you can be put on a low dose of thyroxine just to see if it does make any difference’. I was wondering if that was going to be at all possible.

GP: Yes, I think that’s a difficult one actually.

Here, the patient displaces the responsibility for suggesting thyroxine (a medication used to treat underactive thyroids) from herself to the original speaker, her friend. After this request made through ‘displaced authorship’, the patient raises a specific question in which she compels the GP to give an answer. Such clarifications, however, do not make much difference: the GPs usually recognise the meaning of third-party references anyway. In this case, the GP responds by defining it as ‘difficult’ to answer. After some discussion, the GP offers some blood tests to test her thyroid function. Through the patient record, we learn that the tests revealed abnormal findings, and the patient was offered the requested medication. (For a more extensive analysis of this consultation, see Lian et al., 2023.)

#### References to public sources

A woman in her early 30s who is planning for pregnancy mentions an anti-depressant she has read about in online ‘forums’ in a discussion with her ‘usual’ GP. She worries that her current medication might harm her baby if she becomes pregnant:

Case 3:

P: I’ve read a lot about this thing called trazodone. I don’t even know what that is, what is trazodone? [. . .]

GP: I’ve never – it’s certainly not something that they would routinely use.

P: I know forums, you should stay away from forums but, actually, I find the baby centred ones really quite useful [. . .] actually quite reassuring, like, ‘My baby’s fine’. So, I actually do find them quite helpful.

After quoting from internet forums about the possibility of taking trazodone, the patient downplays her own knowledge (‘this thing’ and ‘what is trazodone?’). After the GP has explained that this is not something that they routinely use, which the patient might interpret as a hesitation or an indirect rejection, the patient takes a defensive stance about her online source by making clear she is aware of the risks of misinformation in online sources (‘I know forums, you should stay away from forums’). By doing so, she avoids challenging the GPs professional expertise with information she has sourced from online forums.

In another consultation, a man in his mid 60s refers to a television programme which taught him what ‘other doctors’ do in relation to bowel problems (so a dual third party: a television programme and doctors):

Case 4:

P: I saw a TV programme about it, which is perhaps dangerous as a little knowledge. They said that some people are diagnosed with Irritable Bowel Syndrome, and other doctors prefer to try and take every effort to find if you’re allergic to something first. My mother had a slow bowel, a lazy bowel. I don’t know whether that might have had any connection.

GP: A lazy bowel is usual constipation, okay.

P: That’s the opposite, then, in effect.

GP: Yes, but again it can be the other end of Irritable Bowel.

By citing a third-party source, the patient sets the normative standard for how doctors ought to act in cases like his, and that is to take an allergy test which could reveal the cause of his symptoms and, therefore, what kind of medical treatment he needs (which would be the main purpose of the test). *Before* doing so, however, he makes sure to confirm his own lay status (‘perhaps dangerous’) and thereby, indirectly, acknowledge the GP’s expertise and superior epistemic position. The GP, who is not his ‘usual’ one, sidesteps the patient’s arguments and does not offer him any test, but instead medication used for irritable bowel symptoms (not allergy related, and not dealing with causal factors). The patient does not seem to gain anything by referring to this source.

### Patients use third-party references as contra-arguments

#### References to ‘displaced author’/‘proxy patient’

When patients use third-party references as contra-arguments, which they usually do, it often relates to concerns about medication side-effects, particularly preventive cardiovascular medication (beta-blockers to reduce blood-pressure and statins to reduce cholesterol), as seen in the next five cases. The following quote is a rare example of a third-party reference accompanied by overt opposition:

Case 5:

P: I’m not taking statins, definitely not.

GP: Oh. Why was that response not unexpected?

P: Well, in as much as a pharmacist friend of mine took them, not only did she have these muscular weaknesses, and she also had occasional diarrhoea. Well, I certainly don’t want that, you know?

Here, the patient, a woman in her mid 80s, opposes statins directly, before justifying her opposition by citing the experiences of a friend with intrinsic authority on these matters. Her ‘usual’ GP acknowledges the patient’s right to decide, and accepts her choice.

In another consultation, a woman in her early 70s, diagnosed with hypertension, is concerned about possible side-effects of her blood-pressure medication:

Case 6:

P: Because he [other doctor] put me on beta blockers, but my friend, my cousin said to me, ‘If you’ve got asthma, or signs of asthma, you shouldn’t be on beta blockers, because they make you breathless’. [. . .]

GP: . . . if I write to him [other doctor] and ask him to see you, would that be okay?

By quoting what her cousin said about side-effects of beta blockers, which she says another doctor ‘put’ her on (not presenting herself as the agent here), she indirectly expresses concerns about her medication. Her ‘usual’ GP interprets it as such and suggests referring her back to the doctor who originally prescribed it, rather than discussing it further.

Similarly, a woman in her late 70s with painful legs is concerned about it being a side-effect of her blood-pressure medication:

Case 7:

P: Someone was talking [with] my friend across the road, who has trouble with her legs, and she said, ‘But everyone I know that has trouble in their legs also is on blood pressure pills’. Is there a connection, do you think?

GP: A direct connection that people that are on blood pressure medication have problems with their legs? I think a third of the population are on blood pressure tablets if they get into their 60s and 70s. And the same group of people start to get arthritis and wear and tear in their joints, so it may be more of a coincidence. I think there are certain medicines, and in particular cholesterol tablets, that can sometimes cause aches and pains in the legs. I think in your case, the symptoms fit very well with a trapped nerve, and I would guess that’s less likely to be due to your blood pressure medication. And I think it will be much more beneficial to us if you keep your blood pressure well controlled to stop and reduce the risk of strokes and heart attacks and things.

After the patient has expressed her scepticism towards blood-pressure medication by quoting what a friend’s acquaintance said about commonly seen side-effects, she asks a direct question: ‘Is there a connection, do you think?’. The patient seems to interpret the long and complex answer from her ‘usual’ GP as a ‘no’ and makes no further objections.

In a discussion with his ‘usual’ GP, a man in his late 70s expresses his concerns about possible side-effects of his blood-pressure medication by referring to a pharmacist:

Case 8:

P: I saw the pharmacist the other day doing my review of medication which they called me in for. She said, ‘You’re on a lot of blood pressure tablets, Name’. I said, ‘Well doctors keep an eye on that’ etc., etc. She said, ‘Hmm, okay’. [. . .]

GP: Unless your kidney function comes back as a little bit more unhappy, at the moment I’d quite like to leave things as they are.

Quoting a source to question his prescribed medication, while also signalling confidence in the GP (‘doctors keep an eye on that’), is a way of questioning without overtly challenging the GPs expertise. The GP interprets this as a request to cut down on his current medication but would ‘quite like to leave things as they are’. The consultation ends with what the GP refers to as a ‘compromise’, which is a wait-and-see strategy: they will await the results of blood tests before deciding what to do. The GP acknowledges the patient’s concerns not by changing his medication, but by ordering blood tests that could support further discussions.

A woman in her late 60s who meets her ‘usual’ GP expresses concerns about possible side-effects of taking blood-pressure medication by quoting a previous dialogue between herself and another person:

Case 9:

P: I actually had a conversation with [name] where I said, ‘Is it worth me taking these tablets and having a longer life presumably because my kidneys are being supported, or packing it in and going back to not being so irritable, being able to sleep and not getting cramp and not being wretched, weak and feeble?’ And she said, ‘Well of course you’ve got to take the tablets, you must live as long as [you] can forever’, you know? And so, I am carrying on, but I cannot say it is really making my life a lot better.

GP: No. Do you think it is the tablets? I understand they do all carry their side effects, but maybe the irritability, do you think you could be depressed?

P: I don’t think I’m depressed; I’ve got nothing to be depressed about.

While quoting her previous dialogue with what appears to be a friend or a relative, she indirectly demonstrates the dilemma she is facing while balancing the pros and cons of taking blood-pressure medication (not telling her GP directly, but what she has told someone else). The GP discounts the patient’s reasonable concerns here regarding the quality versus quantity of life and asks whether she could be mistaking depression for medication side-effects, wich the patient immediately rejects. Still, she agrees to carry on with the medication.

Among cases not related to cardiovascular medication is a consultation with a man in his mid 50s who justifies his opposition to an anti-inflammatory medication with reference to what his wife had experienced:

Case 10:

P: . . . you said, ‘naproxen’.

GP: Yes.

P: Right. I know it’s not professional, but my wife takes naproxen. [. . .] She’s got a frozen shoulder. [. . .] She takes that, but ___. She’s taken that since May, and she’s still got it. If that’s the case, I’m thinking, ‘Well, is that the case for me?’

GP: You won’t know. [. . .] Anti-inflammatories reduce the amount of inflammation and pain that you have, so if you’ve got a sore coccyx, then that is the sort of treatment that anti-inflammatories – there are several of them. [. . .] They really help with muscular and arthritic type pain and anything where there’s any inflammation, [. . .] it may not be a magic cure, but I’m hoping that it will make it easier and speed up the recovery.

P: I’ll try it, yes.

GP: You’ve got nothing to lose, in a way. You don’t want to take it for months on end if it isn’t doing any good-

P: This is it.

GP: But a lot of people find, for joint problems, they work fantastically well. So, the only way of finding out whether you’re going to be one of those people that it helps would be just to give it a go. It’s entirely up to you. I won’t be offended at all if you don’t take them, but if you want to try to see if you can get it better, just give it a bit of a go.

P: Give it a go, yes. Alright.

The GP suggests the anti-inflammatory medication naproxen for back-pain, which the patient is reluctant to accept. Based on the experiences of his wife, who has used it for a frozen shoulder for some time but is still not well (‘she takes that, *but*’ . . .), he questions the efficacy of this drug. After being told that ‘a lot of people’ find naproxen to ‘work fantastically well’, and that he has ‘nothing to lose’ by taking it (thereby ignoring the risk of medication side-effects), the patient accepts the suggested medication.

#### References to public sources

A man in his early 60s refers to something he ‘think[s]’ he has ‘read somewhere’ while arguing against using statins (a cholesterol-lowering medication):

Case 11:

P: Obviously there has been quite a lot of information about the simvastatin, hasn’t there? Whether it’s useful, whether it’s actually achieving what it needs to achieve, and I suppose I’ve picked up on that to some extent. I had a lot of pain up in my shoulders, and also, I think I read somewhere that muscle pain is maybe one of the side effects. So, I thought, ‘Well, I’ll give it a go’.

GP: Yes. Because the simvastatin treats the cholesterol, but not the blood pressure. But they’re related ___because it’s all about trying to reduce your risk of heart attacks and stroke and that kind of event, really. [. . .] If you’re no different off the simvastatin then I’d probably stay on it.

Here, the patient questions the efficacy of statins by citing multiple unspecified sources, prefaced with ‘I think’, before explaining that he suspects it gives him shoulder pain. He then explicitly rules out his sources as justification (‘not because I read lots of information about it’) in favour of personal experiences. His ‘not usual’ GP responds by taking the patient’s perspective and saying what he would have done if he was in the patient’s shoes (‘I’d probably stay on it’), which is indirect advice that recognises the autonomy of the patient, including the right to choose what to do about his medication.

During a consultation with her ‘not usual’ GP, a woman in her late 50s with an underactive thyroid indirectly asks about the possibility of discontinuing her medication by referring to something she has read in a magazine:

Case 12:

P: Well, I was reading in a magazine that somebody changed their diet and their lifestyle and found they didn’t need to take it [thyroxine] anymore. [. . .]

GP: I’m not sure you’ll necessarily be able to stop it altogether. But yes, it would be worth a dose change, yes.

Here, the patient argues against her current medication by citing the case of ‘somebody’ in a magazine (a dual reference which shows that even in media sources we are dealing with ‘proxy patients’). Despite the low pressure this reference entails, the GP suggests a lower dose of current medication. The GP’s offer is also based on the results of the patient*’*s latest blood tests, which indicates she might be ‘overmedicated’.

## Discussion

From previous research, we know that patients often present their views indirectly while justifying their visits (Heritage and Robinson, 2009), explaining their illnesses (Gill and Maynard, 2009), and arguing for and against treatment options (Ainsworth-Vaughn, 1998), but patients’ use of third-party references during option-talk has not yet been systematically studied. This is what we aim to do. After identifying the main patterns across 42 clinical consultations, we reflect on some of the key functions and decision-making implications of this interactional practice in relation to the social context in which it occurs.

### The key functions of third-party references

Patients’ use of third-party references relates to the asymmetrical institutional roles of patients and doctors in the clinical consultation. Most importantly, it allows patients to argue pro-and-contra treatment options in a way that enables them to negotiate their role and their epistemic position within the consultation without ‘overstepping the mark’ by violating what they perceive to be the immanent rules of the game.

#### Arguing pro-and-contra without challenging the GPs’ position in the clinical field

Patients who justify the case they present with reference to third-party sources ‘borrow’ authority, whether experiential or biomedical, from others. In cases of ‘displaced authorship’, where patients voice their argument through a third party, they displace responsibility for proposing or opposing treatment onto others to an even greater extent. While doing so, they go to considerable lengths to avoid staging a direct confrontation between the GP and an external source. Where third-party information appears to contradict the GP, patients defer to the GP for clarification (case 7) or indicate their alignment to the GP (case 8). As if in anticipation of scepticism from their addressee, patients express reservations about friends and relatives as sources of medical or experiential knowledge (case 10), and the reliability of media sources (case 3 and 4). In some cases, patients’ critiques of their sources are accompanied by partial defence; they are helpful despite their weaknesses (case 3). Thereby, they express their experiences, and present themselves as well-informed patients, while also confirming the GPs’ authoritative position in the clinical interaction.

Given the GPs’ institutionally based authority-position in the clinical consultation, this interactional practice is understandable. If patients express arguments for or against treatment options overtly, they may be perceived as transcending the role they are normatively accorded. By not treating themselves as the authoritative source but attributing pro-and-contra arguments to someone else, patients avoid challenging the GPs’ institutional authority and biomedical expertise (Ainsworth-Vaughn, 1998). A key aspect of this interactional practice is therefore that patients manage to side-step a direct ‘me-to-you challenge of the physician’s role’ (Ainsworth-Vaughn, 1998: 181) while arguing for and against treatment options. This indicates that patients perceive that their role affords limited opportunity for directly proposing and opposing treatment options. Or at least that, constrained by their subordinate role, arguments may be more successfully advanced by borrowing authority from someone who substantiates their views.

By mitigating pro-and-contra arguments via third parties, patients perform a difficult balancing act: to claim influence without challenging the institutional and epistemic position of the GPs. Their third-party approach has a dual function: to maintain the hierarchical structure of the clinical encounter, and to execute their right to decision-making influence.

#### Redressing their disadvantaged epistemic position

When patients and GPs discuss treatment options, they also negotiate their epistemic position. Through third-party references, patients redress their disadvantaged epistemic position and present themselves as knowledgeable by invoking a wide range of referents and knowledge-sources. In cases where patients refer to someone who has experienced similar conditions and treatments, they use other people’s experiential knowledge and authority to counter GPs’ professional knowledge. Sometimes, they amplify the experiential perspective by adding their own first-hand experiences as well (case 1, 7 and 11). In cases where patients cite other health professionals, they demonstrate the asymmetry of their knowledge and that their warrant for biomedical knowledge is ‘that they were told it, and therefore are not entitled to treat that knowledge as if it were their own’ (Drew, 1991: 40). By implicating dual sources with different epistemic positions, patients combine the authority of several knowledge sources to cautiously mitigate their move into what could be perceived as the GPs’ authority domain.

The concepts of epistemic injustice, which means a wrong done to someone in their capacity as knower (Carel and Kidd, 2014; Fricker, 2007), might shed some light on the basic mechanisms here. *Testimonial injustice*, which occurs when an individual or group in an epistemic disadvantaged position suffers a deficit in credibility, refers to the ways in which somebody (in this case doctors) may routinely and unfairly dismiss claims of knowledge from people who are not sufficiently like themselves, or who hold a lower position in an established social hierarchy. The cause of testimonial injustice is ‘a prejudice through which the speaker is misjudged and perceived as epistemically lesser’ (Fricker, 2017: 53). *Hermeneutical injustice* occurs in relations of unequal power, when a marginalised individual or group is unable to take part in the construction of mainstream understanding or interpretation, and thus unable adequately to conceptualise or communicate important aspects of their experiences. Patients’ third-party references may address both these potential kinds of injustice: first, by increasing the chances that the patient’s knowledge-claims will be heard and not dismissed by the doctor; and second, by enabling the patient to become critically emboldened in their interpretation of their problems, and their views on proposed solutions. In turn, this might contribute to epistemic justice, which is a constitutive condition of non-domination.

### Implications for the role of patients in decision-making processes

The ways in which patients redress their epistemic disadvantaged position through third-party references have implications for their role in decision-making processes. Decision-making influence, however, will only be granted insofar as the GPs recognise and respond to third-party references as pro-and-contra arguments. We therefore need to consider how the subsequent dialogues unfold.

#### From declaratives to pro-and-contra arguments

On very rare occasions (case 2, 5, 7 and 10), third-party references form part of speech acts that are relatively direct, but usually patients simply quote what someone else has said or experienced, without stating their intentions. By not being explicit about what they want to achieve, patients provide GPs with an option rather than an obligation to respond. Apart from one GP who opts out of this offer (case 4), they all recognise patients’ third-party references the same ways we do: as arguments for and against medical treatments.

After converting declaratives into pro-and-contra arguments, the GPs respond accordingly, either by acceptance, rejection, or a stalling manoeuvre. It is the GPs’ response that makes third-party references function as pro-and-contra arguments. The GPs recognise these utterances as arguments – as we do too – because both they and we know the discursive frame of the clinical consultation (patients are positioned in a subordinate position vis-à-vis the GPs), and the purpose of the interaction (to reach shared decisions about what to do).

This brings us back to the question about the relation between national and institutional forces (Nguyen and Austin, 2018). We interpret our findings as primarily linked to the hierarchically positioned roles of patients and doctors in contemporary biomedical health systems. However, national culture-specific factors are not irrelevant. The indirect and cautious modes of utterances we have seen may be more dominant in the English-speaking sub-culture, from which our sample is drawn, than in other cultures.

#### Agenda-setting and decision-making influence

When patients argue for and against treatment options through third-party references, they rightfully claim a role in the agenda-setting and in the decision-making process. Through these arguments, they provide the GPs with important information about themselves, how they perceive their health problems, what they are concerned about, and what they want to do about it. Thereby, they enable the GPs to target to their specific views and concerns (particularly visible in case 1, 3, 7, 10 and 11).

To what extent do patients’ achievements include decision-making influence? Those who upgrade the strength of their third-party references by expressing overt opposition to a treatment receive full decision-making authority (most clearly in case 5). In other cases, it is difficult to tell. While some patients are offered the medical treatment they propose, others are given explanations for why their proposals are not ideal. When further information is needed, they may be offered a diagnostic test (case 2) or a referral to a specialist (case 3). This means that, in one way or another, most patients achieve something through their third-party references, although it might not be what they initially sought, and even if it is only wait-and-see with a promise of discussion of other options sometime in the future. Final decisions about treatment options, however, seem to be based more on clinical and biomedical arguments than on patients’ arguments (case 1, 2, 4, 7, 8, 10 and 12 in particular), and thereby firmly reside within the GPs’ authority domain.

### Strengths and limitations

Working with observation-data prevents us from asking participants to elaborate their utterances, and our only information about what happens outside the consultation room comes from patient records. Including only 42 cases prevents us from exploring systematic differences between subgroups (such as ‘usual’ GP or not, gender and age). Possible biases in the data relate to recruitment of GPs, who self-selected to take part in the study (Jepson et al., 2017), and participants might have been influenced by their awareness of being filmed. However, our empirical data give us a unique opportunity to explore patients’ use of third-party references in naturally occurring interactions. Observational studies based on audio and video data has been described as ‘the methodological gold standard’ for studying clinical consultations (Timmermans, 2020: 269), insofar as it involves observations of social situations in which interactions are conducted.

## Conclusion

First-hand illness experiences constitute experiential knowledge that gives people with health problems an intrinsic authority and, thereby, an authoritative knowledge-position. When they cross the doorstep to the consultation room and become patients, their experiential knowledge becomes subordinate vis-à-vis clinicians in a hierarchically structured field of interaction. As patients, they face normative expectations about what to say, how to say it, and what not to say. These cultural norms are attached to them through their institutional position in the social field. To understand patients’ interactional practices during decision-making processes, the interactional context is vital.

In this study, we have seen that during option-talk with their GPs, patients often mitigate their pro-and-contra arguments by attributing them to a third party. The ways in which they pose these utterances, and the ways in which the GPs acknowledge and respond to them, show that both parties adhere to the institutional context in which they interact. Most importantly, third-party references allow patients to redress their subordinate and disadvantaged epistemic position by claiming decision-making influence and take part in the decision-making process without being directly confrontational. In addition, it increases patients’ agenda-setting influence (by suggesting what to discuss), and it provides GPs with information that enables them to target their response to patients’ specific views and concerns. In turn, this might limit the risk of patients being wronged in their capacity as knowers and thereby contribute to epistemic justice, which is a constitutive condition of non-domination.

A key rhetorical function of third-party references is to argue pro-and-contra treatment options, which, in turn, creates a kind of safety margin that enables patients to negotiate their role and epistemic position within the consultation, and thereby contribute to SDM, without violating the immanent rules of the game. By adhering to what they perceive to be the discursive opportunities of the clinical field, patients are able to propose and oppose treatment options without threatening GPs’ authority and expertise, which their disadvantaged epistemic position demands. By doing so, they both challenge and maintain the hierarchical structure of the clinical consultation. Although final decisions about treatment options appear to be based more on clinical and biomedical arguments than on patients’ arguments, patients’ use of third-party references might play a key role in safeguarding an essential component of SDM: that decisions are undertaken in collaboration with patients, not just on their behalf.
